# Intimate partner violence norms cluster within households: an observational social network study in rural Honduras

**DOI:** 10.1186/s12889-016-2893-4

**Published:** 2016-03-08

**Authors:** Holly B. Shakya, D. Alex Hughes, Derek Stafford, Nicholas A. Christakis, James H. Fowler, Jay G. Silverman

**Affiliations:** Department of Global Public Health, School of Medicine, University of California San Diego, 9500 Gilman Drive, #0507, La Jolla, CA 92093-0507 USA; Department of Political Science, University of California San Diego, La Jolla, CA USA; Department of Political Science, University of Michigan, Ann Arbor, MI USA; Department of Sociology, Yale University, New Haven, CT USA

**Keywords:** Intimate partner violence, Social norms, Social network analysis, Honduras

## Abstract

**Background:**

Intimate partner violence (IPV) is a complex global problem, not only because it is a human rights issue, but also because it is associated with chronic mental and physical illnesses as well as acute health outcomes related to injuries for women and their children. Attitudes, beliefs, and norms regarding IPV are significantly associated with the likelihood of both IPV experience and perpetration.

**Methods:**

We investigated whether IPV acceptance is correlated across socially connected individuals, whether these correlations differ across types of relationships, and whether social position is associated with the likelihood of accepting IPV. We used sociocentric network data from 831 individuals in rural Honduras to assess the association of IPV acceptance between socially connected individuals across 15 different types of relationships, both within and between households. We also investigated the association between network position and IPV acceptance.

**Results:**

We found that having a social contact that accepts IPV is strongly associated with IPV acceptance among individuals. For women the clustering of IPV acceptance was not significant in between-household relationships, but was concentrated within households. For men, however, while IPV acceptance was strongly clustered within households, men’s acceptance of IPV was also correlated with people with whom they regularly converse, their mothers and their siblings, regardless of household. We also found that IPV was more likely to be accepted by less socially-central individuals, and that the correlation between a social contact’s IPV acceptance was stronger on the periphery, suggesting that, as a norm, it is held on the periphery of the community.

**Conclusion:**

Our results show that differential targeting of individuals and relationships in order to reduce the acceptability and, subsequently, the prevalence of IPV may be most effective. Because IPV norms seem to be strongly held within households, the household is probably the most logical unit to target in order to implement change. This approach would include the possible benefit of a generational effect. Finally, in social contexts in which perpetration of IPV is not socially acceptable, the most effective strategy may be to implement change not at the center but at the periphery of the community.

**Electronic supplementary material:**

The online version of this article (doi:10.1186/s12889-016-2893-4) contains supplementary material, which is available to authorized users.

## Background

Intimate partner violence (IPV), here defined as physical abuse, is a complex global problem, not only because it is a human rights issue, but also because it is associated with chronic mental [1, 2] and physical [3–7] illnesses as well as proximate, acute health effects related to injuries for women [1, 8] and their children [5, 9, 10]. The proportion of partnered women who have ever experienced IPV varies widely across the developing world; with reported rates as low as 12 % in Haiti to as high as 71 % in Bangladesh [11], although differences in these rates may be the result of willingness to report. Reported risk factors are inconsistent across studies, although some common determinants include: poverty, young age, adolescent marriage, low levels of education, patriarchal belief systems, and high levels of alcohol consumption among husbands (12–20). In addition, social support seems to be protective against IPV, although it has been difficult to identify whether women who experience IPV withdraw socially or otherwise face mobility restrictions from abusive partners, or if, instead, social support in and of itself is a source of protection [12].

Attitudes, beliefs, and norms regarding IPV are significantly associated with the likelihood of both IPV experience and perpetration, as well as with willingness to report. Across many contexts, men who believe that IPV is acceptable are more likely to perpetrate IPV, and importantly, women who believe that IPV is acceptable are more likely to report experiencing IPV [11, 13, 14]. Several factors may explain the fact that, for women, accepting IPV is associated with experiencing it. Women who believe that IPV is acceptable may be more likely to enter into relationships with IPV perpetrators, or women who experience IPV may justify their experience by expressing support for IPV perpetration. In fact public health research suggests that violent behavior tends to cluster within families, and there is a rich literature on the “intergenerational transmission” of violence. People who witness IPV in their homes as children are more likely to experience or perpetrate IPV as adults [11–13, 15–20]. Qualitative work in Jordan shows that fathers often encourage sons to perpetrate IPV against their wives as a way of asserting their masculinity [21]. Separate research in Jordan and among Indian immigrants in the US have shown that women are more likely to experience IPV when they are also experiencing violence from their in-laws [19, 22], providing evidence of intra-familial norms. These attitudes and behaviors, while held within families, may also be broadly held within communities, particularly among those with a high rate of overall violence [12].

Given the likely relevance of IPV attitudes for the perpetration and experience of IPV within communities and families, it is important to understand the sources and predictors of these attitudes, as well as to identify how they vary across social contexts. Social norms refer to attitudes and behaviors that are not only prevalent in a society but socially dependent [23]. An individual’s normatively determined behavior is influenced by the behavior of those around her. An important challenge for those who hope to shift normative practices is identification of the *reference group*, or those to whom an individual turns for cues as to what is appropriate or expected [24–26]. Network studies offer valuable insights into norms by demonstrating how the attitudes and behaviors of socially connected individuals are correlated [27–32]. Importantly, network studies can also help identify the types of relationships that are most predictive of shared attitudes and behaviors. In other words, we can use social network analysis to identify reference groups [26, 29, 33]. Finally, using network data, it is possible to evaluate how the structural position of an individual might affect that individual’s behavior in relation to the behavior of others to whom they are connected. *Centrality* measures, for instance, indicate which individuals are most connected within a network, and are often correlated with their ability to influence others, and their tendency to be influenced [34–38].

Although numerous studies have assessed individual attitudes around IPV, none that we know of have mapped IPV attitudes across social networks, allowing for analysis of the relationships within which they are held. In this study, we combine full social network data from two villages in rural Honduras that include assessment of individual acceptance of IPV. Using dyadic level regression analyses, which are a widely used social network method to test correlations between socially connected individuals [38–40], we investigated 1) whether IPV acceptance is correlated across socially connected individuals; and 2) whether these correlations differ across types of relationships (i.e. whether we can identify reference groups). We also used a fundamental measure of network centrality, degree, to test 3) whether social network position is associated with the likelihood of accepting IPV. We hypothesized that IPV acceptance will be most highly correlated across egos and alters with strong social ties such as trust and discussing important matters, and that given the stigma around IPV in Honduras, IPV acceptance will be associated with lower social status.

## Methods

### Data

In 2014, we collected full sociocentric network data from individuals aged 13+ in 2 villages in *La Unión, Lempira*, Honduras. Data were collected as part of a pilot study for a larger intervention project with a focus on maternal and neonatal health in rural Honduras. We included adolescents 13 and above because, in this context, adolescents are likely to form romantic partnerships and have children. Adolescent enrolled at age 13 would have a reasonable chance of giving birth during the course of the larger study. Villages were chosen based on having an adequate size for testing network effects (500+), and for similarity to the demographic characteristics of villages we will be enrolling during the larger study. In each of these villages, we took a complete census of all households, which included mapping each household in the village and enumerating all of the residents within them. We later returned to each household to gather data about individual health indicators, normative beliefs, demographics, and social network connections (see Additional file 1 for details on social network questions). All participants provided verbal consent. Parents of adolescents less than 18 years of age provided additional consent for their children. The Yale IRB and the Honduran Ministry of Health approved all data collection procedures (Protocol # 1405013918) while UCSD IRB approved data analysis for this manuscript (Project # 141622, Exempt).

### Intimate partner violence acceptance

We used 4 questions from the Spanish language version of the Demographic Health Survey for Honduras to assess the conditions under which a person believes that it is acceptable for a man to perpetrate physical violence against his wife or partner. We also extensively tested all survey questions in the population using cognitive interviews before implementing the survey. The questions ask: “*In your opinion, is a husband/companion justified in hitting or beating his wife/companion in the following situations:* (a) *If she leaves the house without telling him?* (b) *Neglects the children?* (c) *Argues with him?* (d) *Burns the food?* Answer choices were either yes or no. We coded a person as positive on IPV acceptance if they answered positively to any of the four questions. Cronbach’s alpha on the full scale was 0.82.

### Social ties

A “name generator” is a question asked of a respondent to help identify important social connections. Our name generators measured family relationships (mother, father, siblings, children, spouse); social relationships (“with whom do you talk”, “with whom do you discuss important matters”, “who do you trust to discuss something personal and private”, “who do you sit with at church”, friends); and support relationships (“who would help if you are sick”; “from whom could you borrow money”; “to whom would you lend money”). (For exact question wording see Additional file 1). For each name generator, respondents (here termed egos) were asked to nominate up to 5 individuals (here termed alters). The type and count of these connections are reported in Table 1. Finally, we created a separate variable to denote whether an ego and a nominated alter were in the same household.

**Table 1:** Summary statistics and breakdown of Ego ~ Alter nominations by name generator

Variable			# of nominations per name generator	
Supports IPV		22 %		
vAge in years (SD)		34 (16)	Mother	350
Gender (Male)		45 %	Father	247
*Religion*			Siblings	874
	Catholic	78 %	Child	212
	Protestant	16 %	Spouse	311
	Other	6 %	Important matters	697
*Education*			Talk	673
	None	44 %	Trust	622
	Primary	41 %	Help when sick	566
	Secondary	14 %	Church	482
	Post-Secondary	1 %	Friend	556
*Marriage*			Borrow	578
	Married	33 %	Lend	544
	Single	29 %	Talk about health	614
	Civil Union	33 %	Community leader	1203
	Separated/Divorced	5 %	Same HH nominated	3051
*Income*			Same HH not nominated	903
	Inadequate, Major problems	12 %		
	Adequate, problems	44 %	Percent of ties same HH by gender	
	Adequate	36 %	Female	31 %
	Adequate & can save	7 %	Male	32 %
Total degree (SD)		15.07 (16)		

### Network structural measures

Sociocentric studies focus on a small population and attempt to ascertain all of the social relationships within a set of interconnected individuals [41]. This is in contrast to *egocentric* network studies that focus on a larger population and attempt to ascertain all of the social relationships of a set of randomly chosen individuals that are usually not connected to one another. Whereas egocentric data may help to improve the representativeness of a sample for a large population, sociocentric data allows measurement of larger network structures (like communities) and individual level network measures based on them. This allows researchers to understand the full extent of the social connections within the community as well as the structure of those connections. Using the igraph library in R, we calculated degree centrality measures for each individual in each village. *Degree* [42] is simply the total number of unique social contacts that nominate or are nominated by the respondent.

### Demographics

We measured a number of individual-level covariates including age, gender, education, income sufficiency, religion and marital status. Our measure of respondents’ education included four categories: (a) No formal education; (b) Primary school; (c) High school; and, (d) University or more. We measured respondents’ income insufficiency according to their responses to the prompt: “*With the total family income, would you say:* (a) *There is enough to live on and save*; (b) *It is sufficient, without major difficulties*; (c) *It is not sufficient and there are difficulties*; or, (d) *It is not sufficient and there are major difficulties*” [43]. Both income and education were included in the models as continuous variables.

### Statistical methods

Our final dataset consisted of one observation for each ego-alter dyad, including pertinent covariates for both individuals. We also created dyads for individuals who lived within the same household, but had not nominated each other in the name generator questions. We used logistic regression to estimate the relationship between individual characteristics and the probability of expressing acceptance of IPV. We corrected for multiple observations of each respondent by clustering standard errors at the individual level using a generalized estimating equation (GEE). Consistent with previous efforts, we assumed an independent correlation structure between the clusters (non-socially connected individuals), which has been shown to be unbiased [44]. The dyadic model provided us the most precision when including covariate information for both the ego and the alter (as opposed to a model with one observation per ego and averaged measures for the alters). Dyads were directed, meaning that, for each observation, we knew who was the nominated alter, and who was the nominating ego. Analyses were performed using R 3.1.2, including the following packages: stargazer, igraph, geepack.

## Results

In total, our household census revealed a population of 1307 individuals. Demographic, normative, and social network data was collected on 831 individuals, who reported 9621 social network relationships.

In Table 1, we report summary statistics on respondents as well as a breakdown of the number of the relationships reported through our name generators. The mean age of respondents was 34 (Range 13–90). Just under 60 % of individuals reported income insufficiency, and 85 % completed no more than primary education. The average number of social connections (degree centrality) per individual across all types of nominated ties was 15.07 (SD 16.61), while for important matters nominations it was 2.00 (SD 1.23) and for trust nominations it was 1.81 (SD 1.02). Approximately 22 % of respondents believed that IPV was acceptable in at least one of the four specified contexts. Approximately 32 % of all nominations (for both men and women) were same household.

### Alter’s IPV acceptance

First, we examined the relationship between social network alters’ IPV acceptance and egos’ IPV acceptance (see Additional file 1: Table S1). Model 1 reports a multivariate model assessing the individual predictors of IPV acceptance. Men and those with higher incomes are less likely to accept IPV than women and those with lower incomes. Older individuals are also slightly more likely to accept IPV. Model 2 reports a bivariate regression between alters’ IPV acceptance and ego’s IPV acceptance of all dyads in our dataset, clustered on egos. We found that, across all social network ties, an ego’s odds of accepting IPV were 2.10 (95 % CI 1.72, 2.55) higher if a social alter accepted IPV. Model 3 adds covariates to Model 2; the results were unchanged. We then analyzed these models further to see if the association between ego’s and alter’s IPV acceptance is gender dependent. Additional file 1: Table S2 shows these results. For women, while there is an indication that opposite gender relationships are more strongly associated, the interaction coefficient in the model is not significant. For men, however, IPV acceptance associations are significantly stronger in opposite gender relationships than they are in same gender relationships.

### IPV acceptance across different categories of alters

**Fig. 1:**
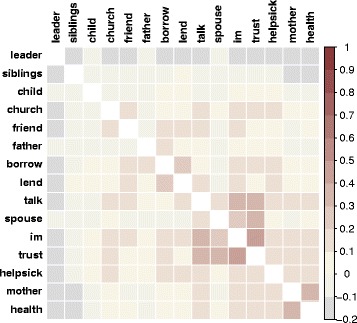
A correlation plot showing the overlap between the nominations made across name generator questions, and ordered according to a hierarchical clustering algorithm. In the top left, there is a cluster of highly overlapping questions, showing that people are likely to nominate the same people as spouses, people with whom they discuss important matters, those they can trust, and those they talk to the most

Given the variety of social relationships in this dataset, we can delve beneath a crude measure of “social connection” to get at the underlying dynamics of relevant social ties. We stratified our data by each type of relationship to determine whether, as predicted, strong social ties were most predictive of correlated IPV acceptance. Table 2 shows these results with adjusted p values using a Bonferroni test for multiple observations. As predicted, the association between ego’s and alter’s IPV acceptance was significant for strong social relationships as well as familial relationships. Those that were not predictive included those nominated as “friends”, borrowing and lending money, as well as “leaders”. While it may seem counter-intuitive that the friend relationship was not significant, previous research has suggested that “friends” as a concept is vague and not predictive of strong ties [45]. Because there was considerable overlap between ties (see Fig. 1 for a correlation plot of overlap between name generators); for instance, an ego could nominate the same alter as a mother and as someone with whom they talk, it was still not possible to determine which ties were the most significant predictors of ego and alter IPV acceptance associations. We therefore created a new set of models (Table 3) in which we created interaction terms for alter’s IPV acceptance by each significant relationship. Including these interactions together in the same model allowed us to estimate the strength of each relationship *conditional on its overlap with other types of relationships*. Model 1 shows that egos were more likely to accept IPV when IPV was accepted by a mother (compared to those with mothers who don’t accept IPV), a father, a spouse, people with whom egos talk regularly, and people whom egos trust to discuss something private.

**Table 2:** Ego-alter IPV concordance, data subset by individual name generators, and adjusted for multiple comparisons

Name generator	Beta	SE	P	Adj P
Mother	1.41	0.29	0	0
Father	1.01	0.39	0.01	0.17
Siblings	0.52	0.22	0.02	0.28
Child	0.65	0.42	0.12	2.06
Spouse	1.25	0.30	0	0.001
Im	0.78	0.21	0	0.004
Talk	1.10	0.20	0	0
Trust	1.10	0.22	0	0
Helpsick	0.32	0.25	0.20	3.32
Church	0.75	0.25	0.002	0.04
Friend	0.18	0.29	0.53	9.04
Borrow	0.30	0.25	0.23	3.84
Lend	0.27	0.25	0.29	4.85
Health	0.72	0.23	0.002	0.03
Leader	−0.06	0.16	0.73	12.40
HH	0.97	0.20	0	0
All	0.64	0.09	0	0

Each row is a separate model with data subset on the name generator indicated

Adj *P* = Bonferroni adjusted p values

**Table 3:** The association of Alter’s IPV acceptance on Ego’s IPV acceptance conditional on relationship overlap

	Model 1 Alter IPV w/all interactions	Model 2 Alter IPV w/all interactions + HH	Model 3 women only	Model 4 men only
Alter IPV	0.35^****^	−0.06	0.12	−0.49^*^
	(0.10)	(0.14)	(0.17)	(0.23)
Non-Nominated In-House Alter* Alter IPV		1.03^***^	1.06^**^	1.26^**^
		(0.25)	(0.33)	(0.38)
Nominated In-House Alter* Alter IPV		1.52^***^	1.64^***^	1.65^***^
		(0.26)	(0.34)	(0.42)
Mother* Alter IPV	0.97^****^	0.55	0.24	1.23^**^
	(0.29)	(0.30)	(0.37)	(0.51)
Father* Alter IPV	0.73^**^	0.39	−0.01	1.06
	(0.36)	(0.37)	(0.48)	(0.61)
Sibling* Alter IPV	0.16	0.33	0.03	1.00^**^
	(0.22)	(0.23)	(0.26)	(0.43)
Spouse* Alter IPV	0.88^***^	−0.20	−0.19	−0.11
	(0.30)	(0.32)	(0.47)	(0.48)
Important Matters* Alter IPV	0.38^*^	−0.07	−0.32	0.38
	(0.21)	(0.21)	(0.28)	(0.33)
Trust* Alter IPV	0.67^***^	0.11	0.08	0.22
	(0.21)	(0.23)	(0.29)	(0.40)
Talk* Alter IPV	0.72^****^	0.25	−0.20	1.07^**^
	(0.19)	(0.20)	(0.25)	(0.36)
Church* Alter IPV	0.40^*^	0.28	−0.02	0.60
	(0.22)	(0.22)	(0.25)	(0.47)
Health Advice* Alter IPV	0.29	0.11	−0.20	0.69
	(0.22)	(0.22)	(0.28)	(0.39)
Income	−0.34^**^	0.32^*^	−0.18^*^	−0.63^**^
	(0.13)	(0.13)	(0.16)	(0.24)
Age	0.01^*^	0.01	0.02	0.00
	(0.01)	(0.01)	(0.01)	(0.01)
Gender Male	−0.55^***^	−0.57^**^		
	(0.20)	(0.21)		
Demographic Controls	Y	Y	Y	Y
Stratify			*Female*	*Male*
Num. obs.	9621	9621	5274	4347
Num. clust.	832	832	449	382

Multiple observations of the same individual adjusted for using GEE. Results of regressions of dependent variable equal to 1 if the subject accepted IPV, 0 otherwise and standard errors reported in parentheses

^****^ *p* < 0.001, ^***^ *p* < 0.01, ^**^ *p* < 0.05, ^*^ *p* < 0.10, complete model with all interaction terms in Additional file 1: Table S3

### IPV acceptance within household relationships

Because many of the relationships most predictive of IPV acceptance were familial and strongly social, we next investigated whether familial relationships were independently predictive of correlated IPV acceptance between egos and alters, or whether these other relationships were actually a proxy for living in the same household (Additional file 1: Table S2 shows the overlap between family and same-household). Model 2 therefore included two additional terms: 1) the interaction between alter’s acceptance of IPV and whether or not ego and alters lived in the same household (for all *nominated* relationships), and an interaction term between alter’s acceptance of IPV and *non-nominated* household relationships (to account for a possible household-effect for household relationships not identified through nominations). Results shows that the inclusion of the household interaction terms eliminated the previously significant association of social alters’ IPV acceptance across other types of relationships, suggesting that the first set of results might have been a proxy for this household effect. The association between ego’s and alter’s acceptance of IPV was higher when IPV was accepted by someone from the same household, and that relationship was even stronger for nominated household relationships. Because “nominated household relationships” are those in which individuals nominated someone with whom they live as also being someone with whom they have a close relationship, the fact that these relationships showed the strongest association is not surprising.

Given the gender roles in the region – men frequently work in the field while women remain at home – we finally examined whether this household relationship might be moderated by egos’ gender. To do so, we estimated two final models that were stratified by gender. In Model 3, we found that, for women, none of the relationship predictors previously identified were significant, while residing in the same household had a significant, substantively strong effect. In Model 4, for men, we also found that alters who accepted IPV and lived in the same household were highly significant predictors of mens’ IPV norms. However men’s IPV acceptance was also associated with the IPV acceptance of their mothers, siblings, and people with whom they talk, independent of household status. Hence for men, as compared to women, several relationships outside of the household were important predictors of IPV acceptance. The significant association for men with the mothers may in part explain our earlier result, which showed that men were more likely to share IPV approval with opposite gender social connections.

**Fig. 2:**
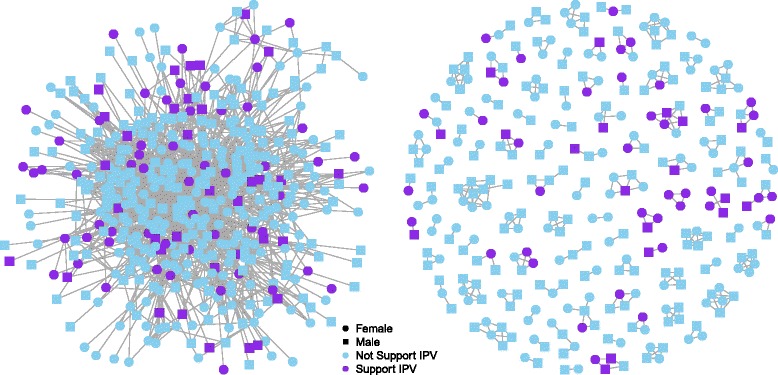
Shows one village’s network from 2 perspectives. The left panel depicts all ties from a randomly selected group of individuals. Note that IPV acceptance is clustered among socially connected individuals and that IPV is generally more accepted on the periphery of the network. The right panel depicts only within household ties from the same randomly selected group. Note the strong clustering of IPV norms at the household level

**Fig. 3:**
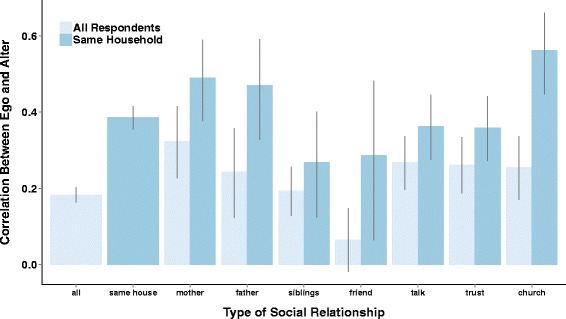
Shows the differential correlation between egos and alter across relationship types depending upon whether or not they live in the same household

Figure 2 shows the clustering of IPV acceptance between connected individuals for a subset of network connections within 1 village, and Fig. 3 depicts the differential correlation between ego’s and alter’s IPV acceptance across relationships depending upon whether or not they live in the same household.

### Network position as a predictor of IPV acceptance

Our final group of models (Table 4) investigates whether degree centrality is associated with IPV acceptance. It could be that subjects who are more socially connected in the community hold different views toward IPV than subjects who are more socially peripheral. In these models, we categorize egos and alters as either well-connected or poorly-connected (above or below the mean individual degree centrality of 15).

**Table 4:** Association between network characteristics and the likelihood that an individual accepts IPV

	Ego degree centrality	Alter IPV* Alter degree centrality
Ego Highly connected	−0.55^**^	
	(0.21)	
Alter Support IPV		0.90
		(0.14)^****^
Alter highly connected		−0.05
		(0.10)
Alter Support IPV* Alter highly connected		−0.55^***^
		(0.20)
Gender male	−0.59^***^	−0.54^**^
	(0.20)	(0.20)
Income	−0.34^***^	−0.33^***^
	(0.13)	(0.13)
Age in years	0.02^**^	0.01
	(0.01)	(0.01)
Education	−0.04	−0.04
	(0.15)	(0.15)
Religion Ref = Catholic		
Evangelical	−0.32	−0.24
	(0.26)	(0.25)
None	−0.03	0.05
	(0.39)	(0.38)
Marital Ref = Married		
Single	0.42	0.45
	(0.32)	(0.32)
Civil Union	0.24	0.29
	(0.25)	(0.25)
Separate or Divorce	0.09	0.20
	(0.43)	(0.44)
Village	0.40	0.31
	(0.19)	(0.20)
Num. obs.	9621	9621
Num. clust.	832	832

Multiple observations of the same individual adjusted for using GEE. Results of regressions of dependent variable equal to 1 if the subject accepted IPV, 0 otherwise and standard errors reported in parentheses

^****^ *p* < 0.001, ^***^ *p* < 0.01, ^**^ *p* < 0.05, ^*^ *p* < 0.10

**Fig. 4:**
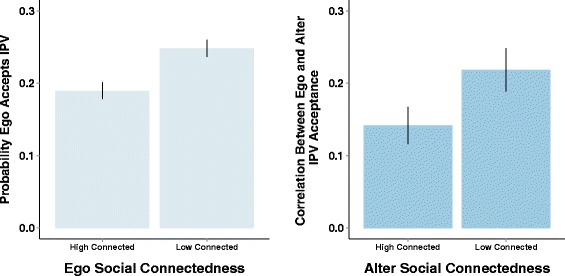
The dynamics around ego and alters network characteristics provide possible clues as to norms. Highly connected egos are less likely to accept IPV (left panel). When alters are poorly connected in the community, the correlation between ego’s and alter’s IPV acceptance is higher (right panel)

In Model 2, we show that there was a negative correlation between individual’s *degree* and their acceptance of IPV. The odds of accepting IPV were 1.73 (95 % CI 1.15, 2.62) times higher if an ego was poorly-connected compared to those who were well-connected, controlling for all demographics. In Model 4, we show that alter’s social position moderated the relationship between ego’s and alter’s IPV acceptance. When alters were well-connected, the strength of the relationship between alters’ IPV beliefs and ego’s IPV beliefs was weaker than when alters were poorly-connected. These results, when taken together (see Fig. 4), suggest that IPV acceptance as a norm is weaker at the center of the social network, and that, instead, these beliefs are both more prevalent and more likely to be shared among those on the periphery.

## Discussion

We analyzed the social network factors that predict IPV acceptance in rural Honduras. Using data from members of the adult and adolescent population, we found that approximately 22 % of people believed that IPV was acceptable. The special contribution of this work is the identification of previously unmeasured social network predictors of IPV norms. First, we found that IPV acceptance was strongly correlated between social contacts. A person was more likely to report acceptance of IPV if another person in his nominated social network also reported acceptance of IPV. Second, these correlations differed across types of contacts. IPV acceptance correlations were strongest between relatives, and those with strong social ties. Third, we found that the correlation between IPV acceptance was strongest for people living within the same household, both those who were nominated as social contacts in our survey and those who were not; this was especially salient for women, for whom the same household relationship seemed to supersede all others. While same household relationships were also salient for men, men’s IPV acceptance was additionally correlated with mothers, siblings, and people with whom they regularly talk, regardless of same household status.

Previous research has provided clues towards a family-level reference group for IPV norms, with multiple studies indicating that women and men who witness IPV in their families as children are more likely to perpetrate or experience IPV as adults [11–13]. Our research is consistent with these previous findings while building upon them using unique social network data. Our results point to the role of families in normalizing and maintaining IPV acceptance- not just for men but for women as well. Furthermore, the very nature of these household, familial ties are intergenerational--not only are peoples’ values on IPV acceptability correlated among same generation family members such as spouses or siblings, but they are strongly correlated with those of parents as well—and, as such, provide an important piece of evidence for intergenerational transmission of IPV accepting norms.

Our structural network analyses suggest that expressing acceptance of IPV is more common on the periphery of the social network, among individuals who are less socially connected. Not only were the people who reported that IPV is acceptable typically less central in the network, the positive relationship between an ego’s and an alter’s acceptance of IPV was stronger when the alter was at the fringe of the network. This is consistent with our own previous work on another social norm in a very different setting (namely latrine ownership in India) [38].

Do these findings point to the fact that IPV norms are primarily held at the fringe of the network, or are those at the fringe of the network more likely to admit finding IPV acceptable? Our results could be impacted by response bias-IPV is a sensitive topic and possibly underreported [46]. Under-reporting may differ depending on what potential social sanction an individual imagines as a result of supporting a stigmatized behavior- reporting itself may then be the consequence of a social norm. Nevertheless, the strong household-level correlations between respondents would suggest that even the possible lack of acceptability of norms supportive of IPV is clustered within closely related groups of co-residential people, which in and of itself is a potentially important clue towards the existence of household-level norms around IPV. There may be, in fact, a dynamic of an “inner norm” and an “outer norm”. The outer norm, or the norm that is openly acceptable within the community, is that IPV is wrong. The reference group, then, for the outer norm, may be the community at large. The inner norm, however, in which co-residential family members may be the reference group, supports the continuation of IPV between generations. Given that the strongest clustering of IPV norms is seen at the household level, it is likely that it is the household and not the greater community that influences the occurrences of IPV in these communities. Perhaps only those on the fringe of the community are ready to violate the “outer norm” and openly express acceptance of IPV, at least in our survey.

### Limitations

There are some limitations to this investigation. First, because of the possibility of a negative outer norm, expressed acceptance of IPV may be underreported. Second we are limited in our interpretations because we do not have reports of actual IPV perpetration. However, because the experience of IPV and attitudinal acceptance of IPV are correlated [11, 13, 14], and because reporting bias exist in both the experience of and acceptance of IPV, it is not clear that measuring experience would change our findings about the social network dynamics of IPV as a norm. Nevertheless, a promising avenue for future research would be the relationship between actual experience or perpetration of IPV with an individuals own perceived acceptability of IPV as well as the perceived acceptability of important others. Third, our data is cross-sectional, so we are only able to study these associations in a single snap-shot of time; as a result we cannot observe time-dependent dynamics. This is an interesting possible avenue for future work that we are pursing. IPV norms may be held within families due to selection, in which adults choose partners similar in their acceptance of IPV as themselves and their parents; this similarity in IPV acceptance is likely a marker for broader norms of gender equity. Or, alternatively, IPV norms may be held due to influence in which a high-risk partner impresses his or her IPV risk upon the other, recreating the patterns of violence from their childhood; it is most likely some combination of both. Only longitudinal research can tease out these dynamics. Fourth, our data is specific to two villages in rural Honduras and may not generalize outside of this context. Like many Latin American countries, Honduras has a culture of “machismo”, where society expects men to be strong and aggressive, particularly in relation to women [47, 48]. Machismo norms often go hand in hand with IPV [48], making machismo cultures particularly appropriate for a network study of IPV. As many Hondurans now emigrate to other countries, it would also be interesting for future work to investigate the role of migration and exposure to other cultural contexts in these dynamics. To our knowledge, ours is the only network study published on IPV norms. It will be crucial for future work to investigate these dynamics in different cultural contexts in order to determine globally applicable social patterns in IPV acceptance. Finally, because of resource constraints, we were only able to survey 64 % of the population.

## Conclusion

Ultimately, this research speaks to the importance of differential targeting of individuals and relationships in order to reduce the acceptability and, subsequently, the prevalence of IPV. Previously suggested norm-change interventions target patriarchal belief systems and social dynamics that normalize violence, and current research points to the importance of working with men in order to change norms around IPV [49, 50]. Attempting to educate an individual man around the negative aspects of IPV may be of little use if he returns to a family in which his father, mother, and brothers perpetrate and promote the same behaviors that he has been taught to eliminate. Our results inform possible strategies for implementing effective interventions. What is most important for interventionists may be that, because IPV norms seem to be strongly held at the household, the household is probably the most logical unit to target in order to implement change. This household level approach would also include the possible benefit of a generational effect. Finally, in social contexts in which perpetration of IPV is not socially acceptable, interventionists might find that the most effective strategy is to implement change not at the center but at the periphery of the community [51].

## Additional file

Additional file 1: Online appendix for: Intimate partner violence norms cluster within households: a sociocentric network study in rural Honduras. (DOCX 146 kb)

## Abbreviations

CI: confidence interval
DHS: Demographic and Health Surveys
IPV: Intimate Partner Violence
OR: odds ratio
SD: standard deviation
