# Supplementary material for: Intimate partner violence norms cluster within households: an observational social network study in rural Honduras
Source: BMC Public Health. 2016 Mar 8;16:233. doi: 10.1186/s12889-016-2893-4 (PMC4782313; doi:10.1186/s12889-016-2893-4)
Supplement: Additional file 1: — Online appendix for: Intimate partner violence norms cluster within households: a sociocentric network study in rural Honduras. (DOCX 146 kb) [file 12889_2016_2893_MOESM1_ESM.docx]

**Online appendix for: Intimate partner violence norms cluster within households: a sociocentric network study in rural Honduras**

**Name generators: We asked each individual the following questions**:

| Does your MOTHER still live in this town? |
| --- |
| What is the name of your MOTHER? |
| Does your FATHER still live in this town? |
| What is the name of your FATHER? |
| Do you have any SIBLINGS living in this town? |
| What are their names? |
| Do you have any CHILDREN who don’t live with you, but do live in this town over the age of 12? |
| What are their names? |
| Are you married or living in a civil union? |
| What is his/her name? |
| On a normal day, who is a person that you spend the most time talking to? |
| What is the name of a person with whom you discuss IMPORTANT MATTERS?  (including friends, family, people you work with, people who work for you, neighbors, etc.) |
| What is the name of a person that you could trust to talk about something personal or private?  (including friends, family, people you work with, people who work for you, neighbors, etc.) |
| If you were sick or unable to work, what is the name of a person who would come to help you care for your family? (including friends, family, people you work with, people who work for you, neighbors, etc.) |
| What is the name of a person with whom you sit at church? (including friends, family, people you work with, people who work for you, neighbors, etc.) |
| What is the name of your best FRIEND? (including friends, family, people you work with, people who work for you, neighbors, etc.) |
| Do you have a PATRON/PATRONA? |
| What is his/her name? |
| Do you work with anyone? |
| What are their names? (including friends, family, people you work with, people who work for you, neighbors, etc.) |
| If you needed 200 Lempira for the day, what is the name of a person from whom you would borrow it? (including friends, family, people you work with, people who work for you, neighbors, etc.) |
| What is the name of a person to which you would lend 200 Lempira for the day? (including friends, family, people you work with, people who work for you, neighbors, etc.) |
| What is the name of a person that you ask for advice about health related matters? (including family, people you work with, people who work for you, neighbors, etc.) |
| What are the names of this town’s leaders? (including friends, family, people you work with, people who work for you, neighbors, etc.) |

For each question respondents were allowed to name up to 5 people. In general people nominated more individuals as leaders than for the other types of relationships. We believe this is because the leader question was worded in the plural. We did not use the work or patron relationships in this analysis as very few nominations were named in either of those categories. While our future work will include photographic identification of nominated individuals, for this data collection effort and consistent with most social network data collection, we did not have that capability.

Table S1: Results of a logistic regression analysis showing individual predictors of acceptance of IPV, rural Honduras

|  | Model 1 | Model 2 | Model 3 |
| --- | --- | --- | --- |
|  | Individual predictors | Alter IPV | Alter IPV + individual predictors |
| Alter IPV |  | 0.74_****_ | 0.64_****_ |
|  |  | (0.10) | (0.09) |
| Gender male | -0.57_**_ |  | -0.54_***_ |
|  | (0.20) |  | (0.20) |
| Income | -0.35_**_ |  | -0.33_**_ |
|  | (0.13) |  | (0.13) |
| Age in years | 0.01 |  | 0.01 |
|  | (0.01) |  | (0.01) |
| Education | -0.05 |  | -0.04 |
|  | (0.15) |  | (0.15) |
| Religion Ref=Catholic |  |  |  |
| Evangelical | -0.26 |  | -0.24 |
|  | (0.25) |  | (0.25) |
| None | 0.09 |  | 0.07 |
|  | (0.37) |  | (0.37) |
| Marital Ref=Married |  |  |  |
| Single | 0.47 |  | 0.45 |
|  | (0.32) |  | (0.32) |
| Civil Union | 0.31 |  | 0.30 |
|  | (0.25) |  | (0.25) |
| Separate or Divorce | 0.19 |  | 0.20 |
|  | (0.43) |  | (0.43) |
| Village | 0.31 |  | 0.28 |
|  | (0.19) |  | (0.19) |
| Num. obs. | 9621 | 9621 | 9621 |
| Num. clust. | 832 | 832 | 832 |
| ^****^p < 0.001, ^***^p < 0.01, ^**^p < 0.05, ^*^p<0.10  Multiple observations of the same individual adjusted for using GEE, Results of regressions of dependent variable equal to 1 if the subject accepted IPV, 0 otherwise and standard errors reported in parentheses. | | | |

Table shows beta coefficients and standard errors ().

Table S2: Results of a logistic regression analysis showing the association between individuals IPV acceptance with that of their social contacts, by same gender relationships, and stratified by gender.

|  | Model 1 | Model 2 |
| --- | --- | --- |
|  | Women only | Men only |
| Alter IPV | 0.54^****^  (0.15) | 1.13^****^  (.21) |
| Alter IPV*Alter gender (male) | 0.32  (0.22) | **-0.98^****^**  **(0.28)** |
| Alter gender male | -0.12  (0.10) | 0.21  (0.13) |
| Income | -0.19  (0.22) | -0.64^***^  (0.24) |
| Age in years | 0.02  (0.01) | 0.00  (0.01) |
| Education | -0.21  (0.18) | 0.28  (0.24) |
| Religion Ref=Catholic |  |  |
| Evangelical | -0.36  (0.31) | -0.01  (0.42 |
| None | 0.59  (0.59) | -0.16  (0.53) |
| Marital Ref=Married |  |  |
| Single | 0.32  (0.38) | 0.50  (0.53) |
| Civil Union | 0.30  (0.30) | 0.43  (0.42) |
| Separate or Divorce | 0.06  (0.49) | 0.72  (1.21) |
| Village | 0.17  (0.24) | 0.55  (0.32) |
| Num. obs. | 5274 | 9621 |
| Num. clust. | 450 | 832 |
| ^****^p < 0.001, ^***^p < 0.01, ^**^p < 0.05, ^*^p<0.10 | | |

Table shows beta coefficients and standard errors ().

Table S3: Proportion of same household and not same household ties by family relationship type

|  | Mother | Father | Sibling | Child | Spouse |
| --- | --- | --- | --- | --- | --- |
| Not same HH | 0.16 | 0.11 | 0.72 | 0.15 | 0 |
| Same HH | 0.24 | 0.17 | 0.21 | 0.05 | 0.38 |
